# Inconsistent Condom Use among Public Primary Care Patients with Tuberculosis in South Africa

**DOI:** 10.1100/2012/501807

**Published:** 2012-07-31

**Authors:** Gladys Matseke, Karl Peltzer, Julia Louw, Pamela Naidoo, Gugu Mchunu, Bomkazi Tutshana

**Affiliations:** ^1^HIV/AIDS, STI, and TB (HAST) Research Programme, Human Sciences Research Council, Pretoria, Private Bag X41, Pretoria 0001, South Africa; ^2^Department of Psychology, University of the Free State, Bloemfontein, South Africa; ^3^Department of Psychology, University of the Western Cape, Cape Town, South Africa

## Abstract

The high rate of HIV infections among tuberculosis (TB) patients in South Africa calls for urgent HIV reduction interventions in this subpopulation. While correct and consistent condom use is one of the effective means of HIV prevention among sexually active people, there is insufficient research on condom use among TB patients in South Africa. The aim of this paper was to determine the prevalence of inconsistent condom use among public primary care TB patients and its associated factors using a sample of 4900 TB patients from a cross-sectional survey in three health districts in South Africa. Results indicated that when asked about their consistency of condom use in the past 3 months, 63.5% of the participants reported that they did not always use condoms. In the multivariable analysis, being married (OR = 1.66; 95% CI 1.25–2.20) or cohabitating or separated, divorced, or widowed (OR = 3.67; 1.85–7.29), lower educational level (OR = 0.66; 0.46–0.94), greater poverty (OR = 1.60; 1.25–2.20), not having HIV status disclosed (OR = 0.34; 0.25–0.48), sexual partner on antiretroviral treatment (OR = 0.38; 0.23–0.60), and partner alcohol use before sex (OR = 1.56; 1.30–1.90) were significantly associated with inconsistent condom use in the past 3 months. The low proportion of consistent condom use among TB patients needs to be improved.

## 1. Introduction

HIV prevalence has gradually increased among newly diagnosed tuberculosis (TB) patients in South Africa, from 44% in 2006 [1] to 60% in 2010 [2]. The high rate of HIV infections among TB patients calls for urgent HIV reduction interventions in this subpopulation. A study by Zachariah et al. [3] has shown that TB patients are prone to risky sexual behaviour including unprotected sex. Since most HIV infections in South Africa are sexually transmitted [2], there is a need to encourage sexual behaviour change among TB patients as an HIV prevention measure [4]. Correct and consistent condom use is one of the effective means of HIV prevention among sexually active people. There is scarcity of information on condom use behaviour among TB patients in South Africa. Talbot et al. [5] found among tuberculosis patients in Botswana that the majority inconsistently had used a condom in their last 10 sexual encounters, among a survey of adult TB patients in Ethiopia 78% had not used condom [6], and among HIV-infected tuberculosis patients in Thailand 42% reported never using condoms at all [7]. An investigation into condom use behaviour and its associated factors is important in assisting to plan for HIV prevention programmes that focus on promoting condom use among TB patients. The aim of this study is to determine the prevalence of inconsistent condom use among TB patients and its associated factors using a sample of 4900 TB public primary care patients from three health districts in South Africa.

## 2. Methods

### 2.1. Participants and Procedures

This is a cross-sectional survey with tuberculosis patients in public primary care clinics in South Africa. Three provinces, in South Africa, with the highest TB caseloads were selected for inclusion in the study. One district in each province (*N* = 3) with the highest TB caseloads was ultimately included. These districts were Siyanda in Northern Cape Province, Nelson Mandela Metro in the Eastern Cape Province, and eThekwini in KwaZulu-Natal Province. Within each of these three study districts, 14 public primary health care facilities were selected on the basis of the highest TB caseloads per clinic (*N* = 42). The type of health facilities were public primary health care clinic or community health centre. All new TB and new retreatment patients were consecutively interviewed within one month of antituberculosis treatment. The interview was conducted by trained external research assistants for a period of 6 months in all 42 clinics in 2011. A health care provider who identified a new TB treatment or retreatment patient (within one month on treatment) and 18 years and above informed the patient about the study and referred the patient for participation if interested. A research assistant asked for permission/consent from patients attending the public primary care facility to participate in the interview. We have received's ethical approval from the Human Sciences Research Council Research Ethics Committee (Protocol REC no.1/16/02/11). The Department of Health in South Africa has also provided approval for this study.

### 2.2. Measures

#### 2.2.1. Socioeconomic Characteristics

 A researcher-designed questionnaire was used to record information on participants' age, gender, educational level, marital status, income, employment status, dwelling characteristics, and residential status.* Poverty* was assessed with 5 items on the availability or nonavailability of shelter, fuel or electricity, clean water, food, and cash income in the past week. Response options ranged from 1  =  “Not one day” to 4  =  “Every day of the week”. Poverty was defined as higher scores on non-availability of essential items. The total score ranged from 5 to 20, 5  =  being low, 6–12  =  medium, and 13–20  =  high poverty. Cronbach alpha for this poverty index was 0.89 in this sample.

#### 2.2.2. Condom Use

It was assessed with the question “How frequently did you use condoms when you had sex with your most recent sexual partner in the past three months?”. Response options were 1  =  always, 2  =  frequently, 3  =  sometimes, and 4  =  never. Inconsistent condom use was defined as not always using a condom with the most recent sexual partner in the past three months. 

#### 2.2.3. The Kessler Psychological Distress Scale (K-10)

It was used to measure global psychological distress, including significant pathology which does not meet formal criteria for a psychiatric illness [8, 9]. This scale measures the following symptoms over the preceding 30 days by asking: ‘‘In the past 30 days, how often did you feel: nervous; so nervous that nothing could calm you down; hopeless; restless or fidgety; so restless that you could not sit still; depressed; that everything was an effort; so sad that nothing could cheer you up; worthless; tired out for no good reason?”. The frequency with which each of these items was experienced was recorded using a five-point likert scale ranging from ‘‘none of the time” to ‘‘all the time”. This score was then summed with increasing scores reflecting an increasing degree of psychological distress. This scale serves to identify individuals who are likely to meet formal definitions for anxiety and/or depressive disorders, as well as to identify individuals with subclinical illness who may not meet formal definitions for a specific disorder [8]. This scale has been validated in HIV positive individuals in South Africa [10]. There was significant agreement between the K-10 and the MINI-defined depressive and anxiety disorders. A receiver operating characteristic (ROC) curve analysis indicated that the K-10 showed agreeable sensitivity and specificity in detecting depression (area under the ROC curve, 0.77), generalized anxiety disorder (0.78), and posttraumatic stress disorder (PTSD) (0.77) [10]. The K-10 scale was used as a binary variable comparing scores of 30 or more or less. The internal reliability coefficient for the K-10 in this study was alpha  =  0.92. 

#### 2.2.4. Alcohol Consumption

The 10-item Alcohol Disorder Identification Test (AUDIT) [11] assesses alcohol consumption level (3 items), symptoms of alcohol dependence (3 items), and problems associated with alcohol use (4 items). Heavy episodic drinking is defined as the consumption of six standard drinks (10 g alcohol) or more on a single occasion [11]. In South Africa, a standard drink is 12 g alcohol. Because AUDIT is reported to be less sensitive at identifying risk drinking in women [12], the cut-off points of binge drinking for women (4 units) were reduced by one unit as compared with men (5 units), as recommended by Freeborn et al. [12]. Responses to items on the AUDIT are rated on a 4-point Likert scale from 0 to 4, for a maximum score of 40 points. Higher AUDIT scores indicate more severe levels of risk; score 8 indicates a tendency to problematic drinking including 8–19 high risk, and 20–40 probable alcohol dependence [11]. The AUDIT has been validated in HIV patients in South Africa showing excellent sensitivity and specificity in detecting MINI-defined dependence/abuse (area under the receiver-operating characteristic curve, 0.96) [13], and among TB and HIV patients in primary care in Zambia demonstrating good discriminatory ability in detecting MINI-defined current AUDs (AUDIT  =  0.98 for women and 0.75 for men) [14]. Cronbach alpha for the AUDIT in this sample was 0.92, indicating excellent reliability. 

#### 2.2.5. Alcohol Use in the Context of Sex

Participants were asked if they and their sexual partner had used alcohol before having sex in the past three months.

#### 2.2.6. Perceived General Health

Participants were asked—in general, would you say your health is excellent, very good, good, fair, or poor? This measure was categorized based on participant response (1  =  very good  =  excellent/very good, 2  =  good, and 3  =  fair/poor).

TB treatment status, HIV status, Sexually Transmitted Infection (STI) history, and antiretroviral treatment were assessed by self-report and from medical information. HIV testing status was assessed by self-report.

## 3. Data Analysis

Data were analyzed using Statistical Package for the Social Sciences (SPSS) for Windows software application programme version 19.0. Frequencies, means, and standard deviations were calculated to describe the sample. Data were checked for normality distribution and outliers. For non-normal distribution, nonparametric tests were used. Associations of inconsistent condom use, sociodemographics, health variables, and alcohol and drug use were identified using logistic regression analyses. Interaction between predictor variables was examined; none of the variables had a variance iInflation factor (VIF) value above 2.5. Following each univariate regression, multivariable regression models were constructed. Independent variables from the univariate analyses were entered into the multivariable model if significant at *P* < 0.05 level. For each model, the *R* ^2^ are presented to describe the amount of variance explained by the multivariable model. Probability below 0.05 was regarded as statistically significant.

## 4. Results

### 4.1. Sample Characteristics and Inconsistent Condom Use

From the total sample (*N* = 4935) included in the study, 35 (0.7%) refused to participate, so the final sample included 4900, 54.5% men and 45.5% women, with a mean age of 36.2 years (SD  =  11.5), range from 18 to 93 years. Almost two-thirds of the participants (65.2%) were between 25 to 44 years old, 84.6% were Black Africans, 72.5% was never married, 27.7% had completed secondary education, and 17% scored high on the poverty index. Regarding health status of the TB patients, 55% were coinfected with HIV, 23.3% were retreatment TB patients, 46.3% rated their health status as fair or poor, and 26.2% had severe psychological distress. Twenty-two percent were on antiretroviral treatment, 63.5% had disclosed their HIV status to their last sexual partner, and 72.8% had a sexual partner with unknown or HIV negative status. Almost one in five of the participants (23.3%) engaged in hazardous or harmful alcohol use, 11.2% had used alcohol before sex in the past three months, and 9.6% reported that their sexual partner had used alcohol before sex in the past three months. When asked about their consistency of condom use in the past 3 months, 63.5% had used condoms inconsistently (see Table 1).

Table 2 shows both the bivariate analysis reporting the unadjusted odds ratios and multivariable analysis reporting the adjusted odds ratios of all possible predictors of inconsistent condom use. The bivariate analysis showed that older age, being married or cohabitating, or separated, divorced, or widowed, lower educational level, greater poverty, being HIV negative, ever having had a sexually transmitted infection (STI), severe psychological stress, not being on ART, sexual partner not on ART, not having HIV status disclosed to their last sexual partner, partner HIV status unknown or negative, alcohol use, alcohol use before sex, and partner alcohol use before sex were associated with inconsistent condom use in the past 3 months. Finally, in the multivariable analysis, being married or cohabitating or separated, divorced or widowed, lower educational level, greater poverty, not having HIV status disclosed, sexual partner on ART, and partner alcohol use before sex were significantly associated with inconsistent condom use in the past 3 months (see Table 2).

## 5. Discussion

The study found high rates of inconsistent condom use, as found in some other studies [5–7], among a large sample of TB patients in South Africa. This finding is alarming given the high rate of HIV and TB coinfection at a national level in South Africa [2]. The dual epidemics of HIV and TB have become a public health priority and is beginning to receive increasing attention from the National Department of Health as specified in the National Strategic Plan 2012–2016 [15]. TB, therefore, cannot be managed as a single disease entity. A comprehensive treatment and prevention programme for TB, HIV, and indeed other comorbid disorders is required in order to meet this public health challenge. In the context of this study, condom use needs to be considered as one of the HIV prevention measures when planning for HIV prevention programmes for TB patients. 

Further, the results of the study suggest that people who were married/cohabitating and those separated/divorced/widowed, with lower education and living in poverty were more likely to report inconsistent condom use compared to their respective counterparts. Similar to the results suggested in this study, other studies showed that lower levels of education were associated with lack of condom use [16, 17]. Lack of condom use by married people in this study is consistent with findings from previous studies [18, 19]. This finding may be because there is some trust built among people in such relationships leading to decisions to use condoms occasionally or stop altogether. Furthermore, it was suggested by study results that people who have not disclosed their HIV status, those whose sexual partner was not on ART, and those whose partner took alcohol before sex, were more likely to report inconsistent condom use. This finding is of concern since nondisclosure of HIV status and alcohol use in the context of sex coupled with inconsistent condom use may lead to further HIV infection [20, 21]. Efforts should also be made for follow-up couple counselling and testing, use of behaviour rehearsal technique to overcome barriers of disclosure, and integrating alcohol use into HIV/AIDS risk reduction interventions.

## 6. Study Limitations 

Caution should be taken when interpreting the results of this study because of certain limitations. As this was a cross-sectional study,causality between the compared variables cannot be concluded. A further limitation was that most variables were assessed by self-report and desirable responses may have been given, in particular regarding condom use.   

## 7. Conclusion

The control of the concomitant HIV and tuberculosis epidemics is one of the greatest challenges facing South Africa. It is essential to link TB and HIV treatment and prevention programmes in situations where both diseases are prevalent to improve the diagnosis, treatment, and outcomes for patients affected by both diseases. The high rates of inconsistent condom use among TB patients found in this study needs to be improved and both male and female condom use should be considered HIV prevention measures when planning for HIV prevention programmes for TB patients. 

## Figures and Tables

**Table 1:** Sample characteristics and inconsistent condom use.

Socioeconomic factors	Total	Inconsistent condom use
	N (%) or M (SD)	N (%) or M (SD)
All	4900	2697 (63.5)
Age		
18–24	643 (13.3)	315 (57.2)
25–34	1841 (38.1)	1004 (60.4)
35–44	1313 (27.1)	742 (63.6)
45 or more	1040 (21.5)	601 (73.1)
Male	1631 (54.5)	1477 (64.8)
Female	2194 (45.5)	1178 (61.9)
Never married	3356 (72.5)	1772 (60.8)
Married/cohabitating	1001 (21.6)	609 (66.8)
Separated/divorced/widowed	275 (5.9)	165 (80.9)
Grade 7 or less	1269 (26.3)	730 (71.8)
Grade 8–11	2213 (45.9)	1219 (61.6)
Grade 12 or more	1336 (27.7)	714 (59.5)

Poverty index		
Low	1617 (35.0)	802 (56.8)
Medium	2227 (48.2)	1287 (64.5)
High	275 (16.8)	454 (72.3)
Black African	4078 (84.6)	2277 (62.9)
Coloured	634 (13.1)	331 (66.6)
Indian, Asian, White, other	111 (2.3)	59 (66.3)

Health status		
*HIV status*		
HIV negative	1759 (36.9)	954 (64.1)
HIV unknown	385 (8.1)	258 (79.6)
HIV positive	2619 (55.0)	1417 (60.6)
Ever STI	329 (7.3)	212 (69.1)
New TB patient	3707 (76.7)	2061 (63.5)
Retreatment TB patient	1128 (23.3)	610 (63.0)

Perceived health status		
Excellent/very good	928 (19.2)	516 (64.3)
Good	1667 (34.5)	916 (62.7)
Fair/poor	2238 (46.3)	1245 (63.8)
Severe psychological distress	1195 (26.2)	709 (67.1)
On ART	906 (22.0)	421 (53.3)
Disclosed HIV status to last partner	2769 (63.5)	1480 (54.9)
Partner with HIV unknown or negative versus positive	3244 (72.8)	2010 (66.6)
Sexual partner on ART	440 (11.1)	189 (44.8)

Alcohol use		
AUDIT		
Abstinent or low risk (score 0–7)	3688 (76.7)	1985 (61.2)
High risk (8–19)	799 (16.6)	470 (68.5)
Probably alcohol dependent (20–40)	321 (6.7)	205 (76.2)
Alcohol before sex in the past 3 months	549 (11.2)	368 (73.6)
Partner alcohol before sex in the past 3 months	408 (9.6)	325 (75.6)

**Table 2:** Association between inconsistent condom use, socioeconomic factors, health status, and alcohol use.

	Crude OR (95% CI)	Adjusted OR^a,b^ (95% CI)
Socioeconomic factors		
Age	1.02 (1.02–1.03)^∗∗∗^	1.00 (0.99–1.01)
Male versus female	1.13 (1.00–1.28)	0.96 (0.75–1.24)
Never married	1.00	1.00
Married/cohabitating	1.30 (1.11–1.52)^∗∗∗^	1.66 (1.25–2.20)^∗∗∗^
Separated/divorced/widowed	2.73 (1.91–3.90)^∗∗∗^	3.67 (1.85–7.29)^∗∗∗^
Grade 7 or less	1.00	1.00
Grade 8–11	0.63 (0.54–0.74)^∗∗∗^	0.72 (0.53–0.99)^∗^
Grade 12 or more	0.58 (0.48–0.69)^∗∗∗^	0.66 (0.46–0.94)^∗∗^

Poverty index		
Low	1.00	1.00
Medium	1.38 (1.20–1.59)^∗∗∗^	1.60 (1.24–2.20)^∗∗∗^
High	1.98 (1.62–2.43)^∗∗∗^	1.54 (1.05–2.25)^∗^
Black African	1.00	—
Coloured	1.17 (0.96–1.43)	
Indian, Asian, White, other	1.16 (0.74–1.81)	

Health status		
HIV positive versus negative	0.86 (0.75–0.99)^∗^	0.96 (0.72–1.28)
Ever STI	1.38 (1.07–177)^∗^	1.10 (0.72–1.67)
New TB versus retreatment	1.02 (0.88–1.19)	—

Perceived health status		
Excellent/very good	1.00	—
Good	0.94 (0.78–1.12)	
Fair/poor	0.99 (0.83–1.17)	
Severe psychological distress	1.26 (1.09–1.47)^∗∗^	1.21 (0.92–1.59)
On ART	0.60 (0.51–0.70)^∗∗∗^	0.79 (0.58–1.08)
Disclosed HIV status to last partner	0.32 (0.28–0.37)^∗∗∗^	0.34 (0.25–0.48)^∗∗∗^
Partner HIV unknown or negative versus positive	1.60 (1.39–1.84)^∗∗∗^	1.18 (0.87–2.12)
Sexual partner on ART	0.41 (0.33–0.50)^∗∗∗^	0.38 (0.23–0.60)^∗∗∗^

Alcohol use		
*AUDIT*		
Abstinent or Low risk (0–7)	1.00	1.00
High risk (8–19)	1.38 (1.16–1.64)^∗∗∗^	1.14 (0.82–1.58)
Probably alcohol dependent (20–40)	2.03 (1.52–2.71)^∗∗∗^	1.44 (0.86–2.42)
Alcohol before sex	1.22 (1.12–1.32)^∗∗∗^	1.08 (0.78–1.50)
Partner alcohol before sex	1.33 (1.21–1.46)^∗∗∗^	1.56 (1.30–1.90)^∗∗∗^

^a^Using “enter” LR selection of variables.

^b^Hosmer and Lemeshow Chi-square 7.16, df 8, 0.516; Cox and Snell *R* ^2^ 0.15; Nagelkerke *R* ^2^ 0.20.

^∗^ *P* < 0.05; ^∗∗^ *P* < 0.01; ^∗∗∗^ *P* < 0.001.
